# Managing Differences Among Pro-nutrition Actors on Corporate Engagement; A Response to the Recent Commentary

**DOI:** 10.34172/ijhpm.2022.7195

**Published:** 2022-03-09

**Authors:** Jessica Fanzo, Yusra Ribhi Shawar, Jeremy Shiffman

**Affiliations:** ^1^Bloomberg School of Public Health, Johns Hopkins University, Baltimore, MD, USA.; ^2^The Paul H. Nitze School of Advanced International Studies, Johns Hopkins University, Washington, DC, USA.; ^3^Berman Institute of Bioethics, Johns Hopkins University, Washington, DC, USA.

 There is much debate about the role of partnerships between civil society and government actors on the one hand, and industries on the other, in achieving better nutrition and health outcomes from food systems. In a recently published commentary, Kraak^1^ expresses concern about fragmentation among the network of actors in civil society and government on corporate-engagement principles and on strategies to strengthen food systems and calls forto to strengthen food systems. She calls for pro-nutrition actors to come together. However, our research findings indicate that the shared vision she calls for, at least in the near-term, is politically unattainable. We instead argue for a more politically feasible strategy: pro-nutrition actors should strive to manage expectations and disagreements so that they can become a more potent political force, partnering with industry where possible, and continuing to challenge industry where necessary.

 Our research shows that for many pro-nutrition actors, even engaging with industry players is off the table. Why is this? Our paper examined factors that impede partnership between public and private sector actors. These include mistrust between civil society/government and private sector actors; a weak architecture for the global governance of nutrition; power imbalances between public and private sector nutrition actors; and a lack of understanding of the causal pathways behind many nutrition problems.

 Reconciling competing visions, harmonizing engagement principles, and bringing forth a shared narrative is difficult when many civil society and public sector actors will not fathom even engaging. In the same way, plenty of private sector actors are not motivated to work with governments and civil society because they have been demonized—in many instances rightly so due to continuing transgressions in public health and environmental stewardship. Similar tensions around the engagement of private or industry actors exist across many other issue areas, including alcohol, climate, tobacco, firearms, and pharmaceuticals.^2–5^

 One such example where engagement was fractured was the United Nations Food Systems Summit (UNFSS) held in September of 2021. The Summit was meant to be a crucial moment to foster a unified road map for global food system transformation. Instead, it was fraught with controversies.^6^ Hundreds of civil society groups, academics, and social movements, many of whom represent indigenous communities and small-scale farmers and producers, boycotted the UNFSS because the agenda was led by a narrow pool of elites and hijacked by corporate interests.^7,8^ This group did not attend or participate in the proceedings leading to the UNFSS, and they instead held another summit at the same time, entitled “The Global People’s Summit on Food Systems.”^9^ A declaration was signed by over 600 organizations and individuals that stated: “[We] reject the ongoing corporate colonization of food systems and food governance under the facade of the United Nations Food Systems Summit … The struggle for sustainable, just, and healthy food systems cannot be unhooked from the realities of the peoples whose rights, knowledge and livelihoods have gone unrecognized and disrespected.”^10^ Accountability was also not considered at the UNFSS in terms of democratic legitimacy and participation^6^ or in terms of monitoring outcomes of commitments made by actors at the Summit.^11^

 Differences exist in the nutrition community, but pro-nutrition actors should not let disagreement on the issue of engaging the corporate sector stand in the way of coming together on other critical issues where disagreements are less stark. After all, the community is, at a deeper level, unified by a profound disquiet concerning global food and nutrition inequities. This shared moral commitment serves as a powerful foundation for nutrition actors to find ways to work together on issues where stances largely align, and to manage differences on issues—such as corporate engagement—where disagreements persist.

## Ethical issues

 Not applicable.

## Competing interests

 Authors declare that they have no competing interests.

## Authors’ contributions

 JF, YRS, and JS contributed equally to the conceptualization and writing of the manuscript.
